# Fixed drug eruption to sitagliptin

**DOI:** 10.1186/s40200-015-0145-2

**Published:** 2015-03-25

**Authors:** Mrinal Gupta, Anish Gupta

**Affiliations:** Sudhaa Skin Centre, Jammu, 180019 India; Acharya Shri Chander College of Medical Sciences, Jammu, 180019 India

**Keywords:** Sitagliptin, Fixed drug eruption, Oral provocation test

## Abstract

Fixed drug eruption is a common adverse effect seen with various drugs notably antibiotics, antiepileptics and non-steroidal anti-inflammatory drugs. Herein we report a case of Sitagliptin induced fixed drug eruption in a 46 year old female who developed circumscribed, erythematous macules all over the body within one week of initiation of Sitagliptin. The lesions resolved with residual hyperpigmentation on cessation of the drug. The diagnosis was confirmed by an oral provocation test which led to a reactivation of the lesions. To the best of our knowledge, this is the first case of fixed drug eruption to Sitagliptin reported in the literature.

## Background

Fixed drug eruption (FDE) is a common cutaneous adverse effect seen with a wide array of drugs like antimicrobials, antiepileptics and non-steroidal anti-inflammatory drugs. FDE is characterized by well circumscribed, erythematous muco-cutaneous macules that can at times develop as early as 30 minutes after exposure to the causative drug, healing with residual hyperpigmentation and recurring at the same site upon subsequent exposure to the same drug. Sitagliptin is a novel antihyperglycemic agent belonging to the class of dipeptidyl peptidase IV inhibitors, which is used as a second line drug for the management of type II diabetes mellitus [1]. Herein we report a case of FDE due to Sitagliptin in a 46 year old female which, to the best of our knowledge, is the first case to be reported in the literature.

## Case presentation

**Figure 1:**
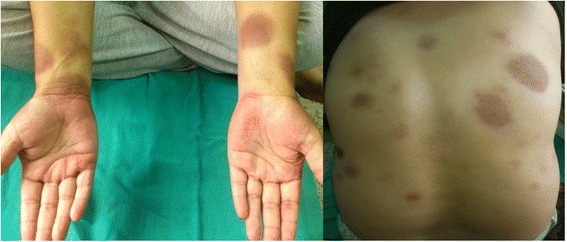
**Fixed drug eruption involving the trunk and extremities.**

**Figure 2:**
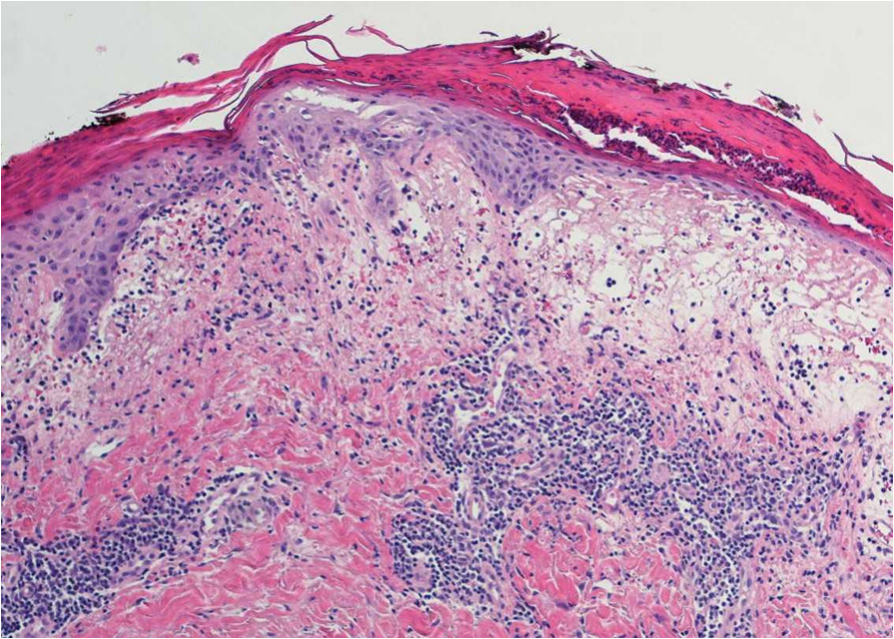
**Histopathology (H&E) showing band like inflammatory infiltrate, perivascular infiltrate and pigment incontinence.**

**Figure 3:**
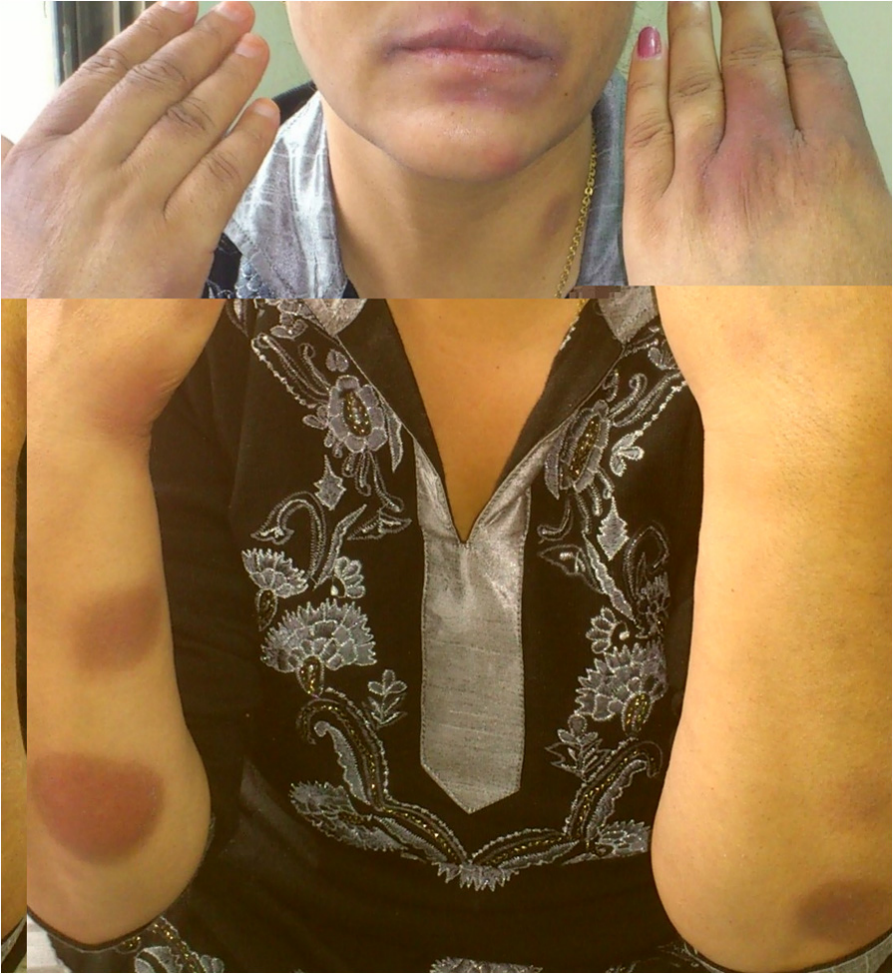
**Increased erythema over pre-existing hyperpigmented lesions after oral provocation test.**

A 46 year-old woman presented in our centre with a three day history of multiple red colored skin lesions which were progressive and were associated with itching and burning sensation. On taking the detailed history, it was revealed that the patient had been suffering from type II diabetes mellitus for the past three years and was being managed with tablet metformin 500 mg twice daily, but one week prior to the onset of skin lesions the patient was also started on tablet Sitagliptin 50 mg/day by her physician in view of the poor glycemic control. After the sixth dose of Sitagliptin, patient noticed multiple circumscribed, reddish lesions over the lips and hands which were associated with burning sensation, which over the next two days progressed to involve the trunk and lower extremities. There was no history of any other drug intake prior to the eruption or any similar lesions in the past. On muco-cutaneous examination, multiple circumscribed erythematous and hyperpigmented round macules were present over the lips, trunk and the extremities whereas the oral and genital mucosae showed the presence of well defined erosions (Figure 1). Nails and hair examination revealed no abnormality. Laboratory tests, including full blood count and biochemistry profile including liver and renal functions, were within normal limits, except for blood glucose, with a value of 167 mg/dl. A skin biopsy was performed and the histopathological examination revealed a dense band like lymphocytic infiltrate, perivascular inflammatory infiltrate, eosinophils and increased pigment incontinence suggestive of fixed drug eruption (Figure 2). At this junction, a diagnosis of FDE was made and all the drugs were discontinued and the patient was started on Prednisolone 40 mg/day and Glimepride. Five days after initiation of oral corticosteroids, the lesions subsided with residual hyperpigmentation. Two weeks later, oral provocation was done, after taking informed consent, and initially metformin was given in full therapeutic dose but no recurrence was observed. After another two weeks, patient was administered Sitagliptin 50 mg and within six hours of administration, there was recurrence of lesions in the form of itching and erythema over the residual pigmented lesions (Figure 3). The patient was again started on a short course of oral corticosteroids and antihistamines which led to clearance of lesions. Causality assessment was carried out using the Naranjo’s scale and the World Health Organization (WHO)‑Uppsala Monitoring centre (UMC) Criteria after which we came to a conclusion that Sitagliptin was the “probable” (Naranjo’s score 6) cause of this adverse drug reaction [2,3]. Keeping in view her medical history and the nature of lesions, a diagnosis of FDE secondary to Sitagliptin was made and the patient was counseled regarding further avoidance of the drug.

## Discussion

Sitagliptin is a newly developed oral hypoglycemic drug for the management of type II diabetes mellitus belonging to the class of dipeptidyl peptidase (DPP)-IV inhibitors, approved by the US Food and Drug Administration in 2006. Apart from systemic adverse effects like hypoglycemia, gastrointestinal effects, pancreatitis, respiratory side effects like nasopharyngitis and upper respiratory tract infections, Sitagliptin has been reported to induce a wide array of cutaneous adverse effects including psoriasiform eruption, maculopapular rash, Stevens Johnson syndrome, toxic epidermal necrolysis, anaphylaxis, cutaneous vasculitis, bullous pemphigoid, photosensitivity and angioedema on co-administration with ACE inhibitors [1,4-7]. A thorough search of literature could not reveal any case of FDE due to Sitagliptin till date.

FDE is a type of delayed hypersensitivity reaction mediated by CD8^+^ T-cells in which the causative drug acts as a hapten, which induces sensitization and development of sensitized CD8^+^ T-cells, which get activated on re-exposure to the offending drug [8]. Although clinical history remains the mainstay of diagnosis, patch tests and drug challenge tests are also helpful and can be used for a more objective diagnostic approach. Patch tests have been found useful in the diagnosis of FDE especially when applied at the previously affected sites. Drug challenge test are the most accurate diagnostic tool for the diagnosis of FDE which can be performed either by starting with a low dose of suspected drug followed by gradual escalation or by starting with the full therapeutic dose at once. A lymphocyte transformation test is a laboratory test for the diagnosis of delayed drug hypersensitivity especially maculopapular drug rash, but have been found useful in FDE also [9]. These tests are of importance, both for the confirmation of diagnosis as well as for the identification of causative drug. Discontinuation of the offending drug forms the mainstay of treatment and may be the only treatment required for mild cases but severe cases may require topical and systemic corticosteroids and antihistamines. Patient education and counseling regarding the avoidance of the offending drug or its derivatives to prevent recurrences constitute an important aspect of management.

## Conclusion

In conclusion, Sitagliptin is a new drug commonly being used for the management of type II diabetes mellitus; henceforth adverse effects caused by it are still not fully known. Cutaneous adverse effects have been reported with Sitagliptin but this is the first case of FDE reported with it. The healthcare providers should be fully aware of the various adverse effects of the drug in order to prevent recurrences and for rapid diagnosis and proper management of the same.

## Consent

Written informed consent was obtained from the patient for publication of this Case report.
